# Therapeutic reference range for duloxetine in the treatment of depression revised: A systematic review and meta-analysis

**DOI:** 10.1016/j.nsa.2024.104077

**Published:** 2024-06-10

**Authors:** F. Amann, M. Kochtyrew, G. Zernig, G. Gründer, X.M. Hart

**Affiliations:** a Central Institute of Mental Health, Department of Molecular Neuroimaging, Medical Faculty Mannheim, University of Heidelberg, Mannheim, Germany; b Medical University of Innsbruck, Department of Pharmacology, Innsbruck, Austria; c Private Practice for Psychotherapy and Court-Certified Witness, Hall in Tirol, Austria; d Arbeitsgemeinschaft für Neuropsychopharmakologie und Pharmakopsychiatrie (AGNP), Working Group "Therapeutic Drug Monitoring", Germany; e Department of Neuropsychiatry, Keio University School of Medicine, Tokyo, Japan; f German Center for Mental Health, partner site Mannheim, Heidelberg, Ulm, Germany

**Keywords:** Duloxetine, Antidepressant, Depression, Reference range, Blood level, Therapeutic drug monitoring

## Abstract

The concentration-effect relationship for the serotonin and norepinephrine reuptake inhibitor duloxetine forms the basis of its therapeutic concentration reference range. However, it lacks systematic investigation and the range reported in the present therapeutic drug monitoring guidelines can only be considered preliminary. A systematic review and meta-analysis were conducted in four databases to determine the optimal target concentration for duloxetine's antidepressant effects and to identify factors that influence its blood concentrations. Relevant articles reporting duloxetine blood concentrations in relation to i) clinical effects/adverse effects, ii) pharmacokinetics and iii) receptor occupancy were analyzed. Out of 340 articles, 11 studies were selected for qualitative synthesis, with seven being included in the quantitative analysis. Three studies showed a positive correlation between duloxetine blood concentrations and antidepressant effects. The adverse event irritability/anxiety was found to be concentration-dependent in one study. Across four studies (n = 223), the 25%–75% interquartile concentration range was 22–72 ng/mL. Two studies reported interquartile ranges of responders (72–116 ng/mL and 65–123 ng/mL), with one of them identifying a threshold concentration for clinical response (58 ng/mL). Neuroimaging studies indicated that 80% serotonin transporter occupancy is reached with blood concentrations above 10–15 ng/mL, while 50% norepinephrine transporter occupancy is observed at 58 ng/mL. We suggest a therapeutic reference range between 20 and 120 ng/mL to achieve optimal antidepressant effects during duloxetine treatment in adults. Due to duloxetine's inhibition of both serotonin and norepinephrine receptors, some patients benefit from low duloxetine concentrations, while others may need higher concentrations to benefit from norepinephrine transporter blockage. In case of non-response at low to medium concentrations (i.e. < 60 ng/mL), we recommend dose titration within the proposed reference range. Blood concentrations can be affected by smoking and certain medications. Hence, therapeutic drug monitoring of duloxetine is strongly recommended for dose titration, particularly with initial prescription.

## List of abbreviations used in the text

BDI: Beck Depression Inventory
C/D: Concentration-Dose ratio
CGI: Clinical Global Impression
CI: Confidence Interval
CYP: Cytochrome P450
d: Day
EC_50_: Effective Concentration associated with 50% transporter occupancy
EC_80_: Effective Concentration associated with 80% transporter occupancy
ED_50_: Effective Dose associated with 50% transporter occupancy
HAMA: Hamilton Rating Scale for Anxiety
HAMD-21: Hamilton Rating Scale for Depression, 21-item version
ICD-10: International Classification of Diseases, 10th Revision
IQR: Interquartile Range
k: Number of studies
MDD: Major Depressive Disorder
mg: Milligram
mL: Milliliter
n: Number of subjects
NET: Norepinephrine Transporter
ng: Nanogram
PET: Positron Emission Tomography
PK/PD: Pharmacokinetic/Pharmacodynamic
PRISMA: Preferred Reporting Items for Systematic Reviews and Meta-Analysis
ROC: Receiver Operating Characteristics
SD: Standard Deviation
SERT: Serotonin Transporter
SNRI: Serotonin, Norepinephrine Reuptake Inhibitor
SPECT: Single Photon Emission Computed Tomography
TDM: Therapeutic Drug Monitoring
UKU: Udvalg for Klinske Undersøgelser side effect rating scale

## 1 Introduction

The selective serotonin, norepinephrine reuptake inhibitor (SNRI) duloxetine is approved for the treatment of major depressive disorder (MDD), generalized anxiety disorder, diabetic peripheral neuropathic pain (Lilly, 2009a), female stress urinary incontinence (Lilly, 2009b), fibromyalgia and chronic musculoskeletal pain (Lilly, 2004) in adults. Duloxetine has a high affinity for the serotonin transporter (SERT) and norepinephrine transporter (NET) and a low affinity for dopamine D_2_, α_1_-and α_2-_adrenergic, muscarinic and histaminergic H_1_-receptors (Bymaster et al., 2001). It is mainly metabolized in the liver via Cytochrome P450 (CYP) 1A2, to a lower extent via CYP2D6, and by Phase II enzymes to pharmacologically inactive metabolites (Lantz et al., 2003; Lobo et al., 2008; Knadler et al., 2011; Kuo et al., 2004). A dose of 60 mg once daily has been shown to be effective for the treatment of depressive disorders (Detke et al., 2002; Bech et al., 2006) and is recommended as starting and maintenance dose (Lilly, 2009a). Dose escalations up to 120 mg per day are considered safe (Wohlreich et al., 2007) and are approved for long-term treatment in responders (Lilly, 2009a) but are not recommended by national and international guidelines for the treatment of depressive disorders (Bundesärztekammer (BÄK) et al., 2022; Kennedy et al., 2016). A review of six randomized, double-blind, placebo-controlled, short-term (8–9 weeks) efficacy studies did not find significantly improved efficacy for duloxetine doses up to 120 mg compared to the 60 mg dose (Bech et al., 2006). Even in case of insufficient response, an advantage of escalating doses beyond 60 mg could not be shown (Brecht et al., 2011; Kornstein et al., 2008). This finding was confirmed by a meta-analysis comparing dose increase to continuation of standard-dose treatment in several antidepressants (Dold et al., 2017). No evidence for the beneficial effect of dose increase after initial non-response was found. However, it is important to highlight that most studies did not measure drug blood concentrations and it is therefore unclear whether therapeutic effective ranges were at all reached (Dold et al., 2017). Blood concentrations of administered duloxetine vary between individuals at a given dose due to interindividual pharmacokinetic variability such as CYP2D6 genotype, gender, age, smoking, ethnicity, and renal and hepatic function (Knadler et al., 2011; Zastrozhin et al., 2020). The blood concentration of duloxetine is consequently a more accurate indicator of the pharmacological activity than the administered dose (Hiemke et al., 2018). National guidelines for the treatment of depressive disorders therefore recommend blood concentration measurements after non-response to initial treatment as basis for dose adjustment decisions (Bundesärztekammer (BÄK) et al., 2022). The relationship between blood concentrations and clinical effects builds the fundamental pharmacologic principle in therapeutic drug monitoring (TDM) and allows dose titration to the desired target concentration range (Hiemke et al., 2018). A target concentration range, hereafter referred to as therapeutic reference range, is proposed by the major international TDM guideline (Hiemke et al., 2018). To attain optimal antidepressant effects while minimizing the risk for adverse effects, for duloxetine a therapeutic reference range of 30–120 ng/mL (laboratory alert level: 240 ng/mL) is currently proposed and drug level guided dosing at the second highest level for special indications or for problem solving is recommended (Hiemke et al., 2018). However, for duloxetine, a concentration-effect relationship has not yet been systematically explored (Eap et al., 2021) and the currently suggested therapeutic reference range should therefore be considered preliminary. The aim of this study was to determine the therapeutic reference range for duloxetine for the treatment of depressive disorders and to discuss the use of TDM for duloxetine in clinical practice. For that purpose, evidence for a relationship between duloxetine blood concentrations and clinical effects/adverse effects was assessed. Secondly, evidence on SERT and NET occupancy from molecular neuroimaging studies was evaluated and moderating factors on duloxetine blood concentrations were identified.

## 2 Material and methods

### 2.1 Literature review and study selection process

We performed a systemic literature review according to the Preferred Reporting Items for Systematic Reviews and Meta-Analyses (PRISMA) statement (Page et al., 2021). Four databases (MEDLINE via the PubMed interface, the Web of Science Core Collection, PsycINFO, and Cochrane Library) were systematically searched according to our previously published protocol (Hart et al., 2021). Search terms for duloxetine, blood concentrations, TDM, positron emission tomography (PET) and single photon emission computed tomography (SPECT) (for full search strings, see Supplement S1) were used for the systematic search. No preset database search filters or restrictions regarding language and publication date were applied. The initial search was carried out in October 2021 and was updated in August 2023 (for the PRISMA flowchart of study selection, see Supplement S2). Reports were screened considering the pre-defined inclusion and exclusion criteria (Supplement Table S3) by two independent reviewers (MK, XMH). Both randomized controlled trials and uncontrolled studies reporting duloxetine blood concentrations as well as reviews and meta-analyses investigating a relationship between duloxetine blood concentrations and clinical effects/adverse effects were included. All studies regardless of duloxetine drug formulation, dosing schemes or design were eligible. All psychiatric indications were included; however, only patients with depressive disorders were considered a representative patient sample in terms of study outcome. Relevant papers were checked for eligibility in full text. In cases where eligibility could not be determined based on the abstract, the full article was reviewed. Any disagreements between the two reviewers were resolved in a subsequent discussion. Our review protocol was registered in the international prospective register of systematic reviews PROSPERO (CRD42021258633).

### 2.2 Qualitative and quantitative synthesis

All studies reporting duloxetine blood concentrations in humans (serum or plasma), also referred to as blood levels, were identified; particularly those for which an association between duloxetine blood levels and clinical effects/adverse effects was found. Considered reports could be qualitative or quantitative, continuous or categorical, but required a structured clinical assessment using a medical rating scale. Secondly, all studies that reported duloxetine blood levels in relation to pharmacokinetics (hereafter referred to as “concentration studies”) were eligible for inclusion and factors influencing duloxetine blood levels in patients were extracted. Thirdly, molecular imaging studies that reported SERT and NET occupancy in relation to duloxetine blood levels including half-maximum effective concentration values (EC_50_) were assessed. For qualitative and quantitative synthesis, the following information was extracted independently by two reviewers (FA, XMH) from the selected articles and compared afterwards: lead author, year, title, country, study design, number and details of subjects, diagnosis, clinical effect or adverse effect measures, main outcomes and mean dose ± standard deviations (SDs) (Supplement Tables S4–S5). In addition, means, SDs, medians, and interquartile ranges (IQRs) of the relevant blood levels, blood level range, and concentration-dose (C/D) ratios were assessed for the entire patient cohort treated with duloxetine as well as for responders and non-responders, where applicable. Data were either extracted from the manuscript or calculated manually when sufficient data was given. In cases where drug blood level data were incomplete, the corresponding authors of eligible studies were contacted for additional data.

### 2.3 Quality assessment of relevant studies and level of evidence

A previously published rating instrument was used by two reviewers (FA, XMH) to independently assess the quality and reporting of TDM components for all included studies and to assess the overall quality of the cohort and cross-sectional studies (Hart et al., 2021) (Table 1; Supplement Tables S6–S8). Any disagreement was resolved through discussion. A level of evidence for a relationship between duloxetine blood levels and clinical effects/adverse effects was found by consensus discussion among all authors as described in our study protocol (Hart et al., 2021; Hasan et al., 2019).

**Table 1:** Level of evidence; Summarized results of the qualitative synthesis. Studies reporting a relationship between duloxetine blood levels and clinical effects/adverse effects.

Reference	Study design and subjects	Concentration-effect relationship	Concentration-adverse effect relationship	PD comed^a^	TDM score	Study score	Comment
De Donatis et al., 2019	Prospective CS with fixed doses (60 mg/d); MDD; *n* = 66	Positive (HAMD-21)	N/A	No	9/10	7/10	Curvilinear (quadratic) relationship between duloxetine blood levels and antidepressant response. Maximum antidepressant effect between 35 and 120 ng/mL
Rovera et al., 2016	Prospective CS with flexible doses (mean 39.7 mg/d at baseline; 61.42 mg/d at 12 months); MDD; *n* = 35	Not found (HAMD-21) Negative (HAMA, BDI)	Not found (checklist for adverse effects; not specified)	No	7/10	5/10	Patients aged ≥65 years.Significant negative correlation between DLX BLs and the percentage of improvement of BDI- (*p <* 0.001) and HAMA scores (*p <* 0.05) at 12 months.
Volonteri et al., 2010	Prospective CS with flexible doses (mean 58 mg/d); MDD; *n* = 45	Positive (HAMA)	Positive (checklist for adverse effects; not specified)	No	8/10	7/10	Curvilinear (quadratic) relationship between duloxetine blood levels and antidepressant response (HAMA) with optimal efficacy between between 40 and 100 ng/mL; no correlation between duloxetine blood levels and HAMD-21 scores. Significant association between the occurrence of irritability/anxiety and higher duloxetine blood levels; CAVE: high drop-out rate
Waldschmitt et al., 2009	Retrospective CSS with flexible doses (mean 80 mg/d); MDD with affective disorder; *n* = 103	Positive (CGI)	Not found (UKU rating scale)	Yes	7/10	5/8	In patients under duloxetine monotherapy (*n* = 36), higher blood levels were identified in responders with CGI = 1 compared to non-responders with CGI ≥2 (threshold found for antidepressant effect at blood levels ≥58 ng/mL)

CGI = Clinical Global Impression; CS = Cohort Study; CSS = Cross-sectional study; d = day; HAMA = Hamilton Rating Scale for Anxiety; HAMD-21 = Hamilton Rating Scale for Depression, 21-item version; MDD = Major Depressive Disorder; mg = milligram; mL = milliliter; n = number of subjects treated with duloxetine; N/A = Not Available; ng = nanogram; PD comed = Concomitant psychotropic medication with antidepressant efficacy; TDM = Therapeutic Drug Monitoring; UKU = Udvalg for Klinske Undersøgelser.

a except for benzodiazepines.

### 2.4 Statistical analysis

A combined meta-analysis was performed using the software R (version 4.0.3) “metafor” and “meta” package. *I*^2^ statistics was used to evaluate the heterogeneity of the studies, with *I*^2^ values > 50% indicating heterogeneity. 95% confidence intervals (CIs) were calculated from mean blood levels, and data were combined using random-effect models based on the *I*^2^ statistic. Five quality assessment criteria that could have a potential influence on the clinical validity of a reference range were identified a priori (Q2b “diagnosis depressive disorder”, Q3a “psychiatric comedication”, Q3b “CYP-interfering comedication”, and Q4 “dose design” and Q6b “sampling at trough”). Their impact as moderating factors on mean blood levels was investigated by subgroup analyses of studies rated sufficient or insufficient on these criteria. Subgroup comparisons were conducted if a minimum of two records per subgroup were available. Forest plots of subgroup differences identified as significant (*p* ≤ 0.05) were retrieved for visualization of subgroup differences. Linear regression analysis was used to display the relationship between duloxetine dose and blood levels.

## 3 Results

### 3.1 Study overview

In total, 340 articles excluding duplicates were identified through database search, of which 246 papers were rejected after reviewing the title and/or abstract. A further 83 papers were excluded after full text screening, resulting in the inclusion of a total of 11 articles (for PRISMA Flowchart, see Supplement S2; for study details, see Supplement Tables S4–S5). Initially, 12 studies met the inclusion criteria and were suitable for a qualitative synthesis. Of these, five concentration-effect studies, four concentration studies, and three neuroimaging studies were identified. As one concentration-effect study reported contradicting information and clarification could not be achieved, this study was excluded during qualitative assessment. Diagnoses varied among studies and included patients with MDD, depressed patients with affective disorders (F31–F34), and different psychiatric diagnoses according to International Classification of Diseases, 10th Revision (ICD-10) criteria that were not further specified (Supplement Tables S4–S5). General quality criteria for the TDM component were assessed for all 11 studies according to our previously published protocol (Hart et al., 2021) as shown in Table 1 and Supplement Table S6. The most frequently missed criterion was “frequent blood measurements” (Q7a, *k* = 7), whereas “sufficiently broad concentration range” (Q7b, *k* = 1) was sufficient in most studies. The second most frequently missed criterion was “dose design” (Q4, *k* = 6) due to flexible dosing in naturalistic settings. “Representativeness of the patient sample” (Q1, *k* *=* 5) was not met mainly due to inclusion of healthy subjects in all three neuroimaging studies but also because one concentration-effect study comprised only patients of 65 years and older and one concentration study lacked a specification of the patient sample. “Diagnosis” (Q2) was rated as insufficient in six studies, primarily because of a heterogeneous patient sample (Q2b, *k* = 4), in the second place because psychiatric classifications and associated classification systems were not reported (Q2a, *k* = 3). “Comedication” (Q3, *k* = 3) was rated as insufficient in two studies because of missing information and in one study because of the co-administration of fluvoxamine. The criterion “analytical method” (Q5) was rated insufficient in one study due to missing information regarding the limit of quantification. “Steady state” (Q6a) was not fulfilled in one of the three imaging studies, whereas “sampling time” (Q6b) was rated insufficient in one concentration study. Overall, a high quality of TDM was found in most studies. Quality for cohort and cross-sectional studies was assessed by means of study type specific scores. For cohort studies, scores ranged from 5 to 9 (with a possible maximum score of 10), whereas both cross-sectional studies scored 5 out of 8 maximum possible points (Supplement Tables S7–S8).

### 3.2 Relationship between blood levels and antidepressant effects after oral duloxetine administration

Three of the four identified concentration-effect studies reported a positive relationship between duloxetine blood levels and antidepressant effects (De Donatis et al., 2019; Waldschmitt et al., 2009; Volonteri et al., 2010), whereas the fourth study showed a significant negative correlation between duloxetine blood levels and the percentage improvement of anxiety symptoms as well as patient's perception of disease severity (Rovera et al., 2016) (Table 1 and Supplement Table S4). A prospective cohort study by De Donatis and colleagues (De Donatis et al., 2019) used a fixed dosing regimen in adult outpatients diagnosed with MDD who were followed for three months in a naturalistic setting (TDM score: 9/10; study score: 7/10). A bell-shaped quadratic function was found to best describe the association between duloxetine blood levels and antidepressant response (percentage improvement of Hamilton Rating Scale for Depression, 21-item version (HAMD-21) score) at one and three months after excluding patients with blood levels lower than 35 ng/mL. A similar association strength as for the quadratic function was found between blood levels and antidepressant response within the currently recommended blood level range of 30–120 ng/mL at three months (Hiemke et al., 2018; De Donatis et al., 2019). The authors therefore suggested that blood levels higher than 35 ng/mL to a maximum of 120 ng/mL might be needed to obtain the maximum antidepressant effect, whereas blood levels higher than 120 ng/mL were not associated with a better antidepressant effect (De Donatis et al., 2019). The naturalistic TDM survey by Waldschmitt and colleagues (Waldschmitt et al., 2009) introduced a considerable amount of bias by including data from adult depressed patients with a broad range of affective disorders (ICD-10 F31–F34) as well as defining clinical global impression (CGI) scores of ≥2 (much improved) as non-response versus CGI = 1 (very much improved) as response (TDM score: 7/10; study score: 5/8). In patients under duloxetine monotherapy with flexible doses, blood levels were significantly higher (*p* < 0.05) in responders (CGI = 1) (median 93 ng/mL, IQR 65–123 ng/mL) than in non-responders (CGI ≥2) (median 47 ng/mL, IQR 23–54 ng/mL) with similar duloxetine doses (76 vs. 83 mg/day). Receiver operating characteristics (ROC) analysis revealed a threshold concentration of 58 ng/mL (*p* *=* 0.011) for clinical response (Waldschmitt et al., 2009). The naturalistic cohort study in adults by Volonteri and colleagues (Volonteri et al., 2010) defined antidepressant response as a 50% reduction in the HAMD-21 total score from baseline. No significant correlation was found between HAMD-21 scores and blood levels (*R*^*2*^ = 0.05; *p* *=* 0.502), whereas a significant curvilinear quadratic relationship (*R*^*2*^ *=* 0.27; *p* = 0.02) between blood levels and clinical improvement on anxiety symptoms (Hamilton Rating Scale for Anxiety, HAMA) with an optimal anxiolytic effect at duloxetine blood levels between 40 and 100 ng/mL was observed (Volonteri et al., 2010). A limitation of the study was the high drop-out rate of 33%, mainly due to adverse effects and treatment inefficacy (TDM score: 8/10; study score: 7/10) (Volonteri et al., 2010). Rovera and colleagues (Rovera et al., 2016) conducted a cohort study with flexible doses in elderly (≥65 years) outpatients. A significant negative correlation between duloxetine blood levels and the percentage improvement of anxiety symptoms (HAMA) as well as patient's perception of disease severity (Beck Depression Inventory, BDI) was observed after 12 months. The authors suggest that this might be due to the high occurrence of adverse effects (34% drop-outs due to adverse effects) (Rovera et al., 2016).

There is little evidence for a relationship between blood levels and antidepressant effects. From the three discussed studies that found evidence for such a relationship, two were prospective cohort studies and one a retrospective cross-sectional study. Hence, a low level of evidence was assigned (Level C).

### 3.3 Relationship between blood levels and adverse effects after oral duloxetine administration

Three studies assessed adverse effects in patients with depressive disorders treated with duloxetine; one of them found a significant association between adverse effects and blood levels (Volonteri et al., 2010), while two studies failed to show a concentration-adverse effect relationship (Waldschmitt et al., 2009; Rovera et al., 2016) (Supplement Table S4). Noteworthy, Waldschmitt and colleagues (Waldschmitt et al., 2009) performed a cross-sectional study and found that only four patients in the monotherapy group (*n* = 36) showed mild adverse effects (assessed by the Udvalg for Klinske Undersøgelser side effect rating scale, UKU), which were associated with a mean blood level of 60 ng/mL (vs. mean blood level of 56 ng/mL in patients with no adverse effects). Due to low blood levels associated with mild adverse effects, an upper threshold level, indicating decreasing tolerability, was not found and the 75th percentile of blood levels in responders (123 ng/mL) was recommended as the preliminary upper threshold level by the authors (Waldschmitt et al., 2009). The cohort study by Volonteri and colleagues (Volonteri et al., 2010) showed a significant association between the occurrence of irritability/anxiety and the highest duloxetine blood levels (measured blood level range: 5–135 ng/mL) compared to the occurrence of nausea, dry mouth/constipation, hot flushes/headache, and dizziness, which were associated with lower duloxetine blood levels. The authors suggest that the occurrence of adverse effects may diminish the beneficial clinical effects of increasing blood levels (Volonteri et al., 2010).

Overall, there is not enough evidence to support a strong relationship between duloxetine blood levels and adverse effects. Only one prospective cohort study found the occurrence of the adverse effect irritability/anxiety at higher duloxetine blood levels and hence a low grade of evidence (Level C) was assigned.

### 3.4 Relationship between blood levels and SERT/NET occupancy

Three studies were identified that investigated duloxetine blood levels in relation to SERT (*k* = 2, *n* *=* 25) or NET occupancy (*k* = 1*, n* *=* 8) in the human brain (Table 2 and Supplement Table S5). Takano and colleagues (Takano et al., 2006) investigated the SERT occupancy in fifteen healthy Japanese adult males. Single oral doses of 5, 20, 40 or 60 mg duloxetine were administered to twelve volunteers; and 60 mg daily were administered for 7 days to three volunteers. Multiple PET scans with [^11^C]DASB were performed. After single administration, the mean SERT occupancies in the thalamus were 43.6 ± 8.8% at 5 mg, 71.3 ± 5.3% at 20 mg, 80.6 ± 4.8% at 40 mg, and 81.8 ± 4.3% at 60 mg. A relationship (*r* = 0.91) between SERT occupancy and duloxetine blood levels was shown. The dose required for 50% SERT occupancy (ED_50_) and the corresponding blood concentration (EC_50_) were calculated as 7.9 mg and 3.7 ng/mL, respectively. This indicates that blood levels above 15 ng/mL would be necessary to obtain 80% of SERT occupancy (Takano et al., 2006) which has been associated with an antidepressant effect (Meyer et al., 2001). After multiple doses of 60 mg, high SERT occupancy levels were maintained despite decreased blood levels (Takano et al., 2006). Based on single-dose SERT occupancy data, Abanades and colleagues (Abanades et al., 2011) developed an approach to predict repeat-dose SERT occupancy. Several PET scans with [^11^C]DASB were performed with ten healthy male adults that were first administered a single dose of 20 mg duloxetine followed by a 20 mg dose on four consecutive days. Approximately 6 h after administration of the last repeated dose, an average occupancy (across the midbrain, striatum and thalamus) of 78 ± 5% was measured with a mean duloxetine blood level of 19.74 ± 7.8 ng/mL. An indirect pharmacokinetic/pharmacodynamic (PK/PD) model estimated an EC_50_ concentration of 2.62 ± 0.93 ng/mL after single dosing (Abanades et al., 2011). Based on this value, an effective concentration associated with 80% transporter occupancy (EC_80_) of 10.5 ng/mL could be calculated. NET occupancy after duloxetine administration was assessed in eight healthy Japanese adult males in a single dose PET study (20 mg, 40 mg or 60 mg) by Moriguchi and colleagues (Moriguchi et al., 2017). (S,S)-[^18^F] FMeNER-D_2_ was used as radioligand to perform one PET scan before and one PET scan 6 h after oral administration of duloxetine. Mean NET occupancies by duloxetine were 32.6 ± 9.8% with an ED_50_ estimated at 76.8 mg. The authors showed a significant correlation between blood levels (mean 29.44 ± 24 ng/mL) and NET occupancy (*r* = 0.72, *p* = 0.044) with EC_50_ estimated at 58.0 ng/mL (Moriguchi et al., 2017) which is based on the association of 50% NET occupancy with an antidepressant effect (Sekine et al., 2010). Comparing EC_50_-values of 3.7 ng/mL (Takano et al., 2006) and 2.62 ± 0.93 ng/mL (Abanades et al., 2011) for the SERT to the EC_50_-value of 58.0 ng/mL (Moriguchi et al., 2017) for the NET, the EC_50_-value for the NET is around 10 to 20 times higher than the EC_50_-value for the SERT. This indicates that higher drug levels are required for sufficient NET occupancy compared to SERT occupancy, which is likely due to a lower affinity of duloxetine for the NET than for the SERT (Moriguchi et al., 2017). To sum up, two PET studies provided data to estimate a lower therapeutic threshold of 10–15 ng/mL relating to 80% SERT occupancy, whereas one PET study suggests that duloxetine blood levels above 58 ng/mL are required to reach optimal efficacy by NET occupancy.

**Table 2:** Selected SERT/NET receptor occupancy studies that report a relationship between duloxetine blood levels or dose and SERT/NET occupancy. EC_80_ estimated from EC_50_.

Reference	Design and subjects	PET tracer	Mean duloxetine dose (range) [mg/day]	Mean duloxetine blood level (range) [ng/mL]	Mean transporter occupancy (%)	EC_50_ [ng/mL]	EC_80_ [ng/mL]	Brain region
Takano et al., 2006	CS; *n* = 15; healthy volunteers; mean age 24.1 ± 2.4; 100% males	[^11^C]DASB	31.3 ± 21.7 (5–60) (after single dosing)	N/A	69.3 ± 16.5 (after single dosing)	3.7 (after single dosing)	15.0 (after single dosing)	thalamus
Abanades et al., 2011	CS; *n* = 10; healthy volunteers; mean age 40.2 ± 11.1; 100% males	[^11^C]DASB	20.0	19.74 ± 7.8 (5.98–34.17)	78.2 ± 5.1	2.6 ± 0.93	10.5	midbrain, striatum, thalamus
Moriguchi et al., 2017	CS; *n* = 8; healthy volunteers; mean age 25.5 ± 5.9; 100% males	(S,S)-[^18^F]FMeNER-D_2_	37.5 ± 16.7 (20–60)	29.44 ± 24 (4.1–80.8)	32.6 ± 9.8	58.0	N/A	thalamus

CS = Cohort Study; EC_50_ = Effective Concentration associated with 50% transporter occupancy; EC_80_ = Effective Concentration associated with 80% transporter occupancy; mg = milligram; mL = milliliter; n = Number of subjects treated with duloxetine; N/A = Not Available; NET = Norepinephrine Transporter; ng = nanogram; PET = Positron Emission Tomography; SERT = Serotonin Transporter.

### 3.5 Pharmacokinetic factors influencing duloxetine blood levels

Eight studies were identified that reported duloxetine blood levels and the influence of potential moderating factors (Table 3).

**Table 3:** Factors influencing duloxetine blood levels after oral administration.

Reference	Dose	Sex	Age	Smoking	Comedication
Waldschmitt et al., 2009	**X**^**1**^	−^7^	−^12^		**X**^**17**^
Volonteri et al., 2010	X^2^	−^8^			
Rovera et al., 2016	−^3^	−^9^	−^13^		
De Donatis et al., 2019		−^10^	−^14^		
Paulzen et al., 2016	**X**^**4**^				
Paulzen et al., 2011	**X**^**5**^	−^11^			**X**^**18**^
Fric et al., 2008	**X**^**6**^			X^15^	
Augustin et al., 2018				**X**^**16**^	

X = correlation found; - = no correlation found; blank = not reported; bold = clinically relevant (*p* < 0.05).

**Dose:**

^1^Significant correlation (Spearman rho) between daily doses and duloxetine blood levels (*r* = 0.24; *p* < 0.01).

^2^Trend for significant correlation between duloxetine dose and duloxetine blood levels (*p* = 0.069).

^3^No significant correlation between duloxetine dose and duloxetine blood levels found.

^4^Moderate but significant correlation (Spearman rho) between daily dose and duloxetine blood levels (*r* = 0.47; *p* = 0.04).

^5^Under monotherapy: significant increase of duloxetine blood levels with increasing duloxetine dose (*p* < 0.001); under co-medication with fluvoxamine: significant increase of C/D ratio with increasing dose (*p* < 0.001).

^6^Linear correlation between dose and duloxetine blood levels at T1 (*r* = 0.47, *p* = 0.01).

**Sex:**

^7,8,9,10,11^No significant difference between males and females regarding mean blood levels.

**Age:**

^12^No significant correlation between duloxetine blood levels and age (*p* = 0.893).

^13,14^No significant correlation between duloxetine blood levels and age.

**Smoking:**

^15^Lower duloxetine blood levels and C/D ratio in smokers compared to non-smokers.

^16^Significantly lower median duloxetine blood levels (38.4% lower; *p* = 0.002) and significantly lower C/D ratios (53.6% lower; *p* < 0.001) in smokers compared to non-smokers.

**Comedication:**

^17^Log-linear mixed model found significantly lower C/D ratios after treatment with lithium, clozapine and mirtazapine (*p* < 0.05) compared to duloxetine monotherapy.

^18^3.7-fold (statistically significant) increase of C/D ratios under co-administering 25 mg fluvoxamine.

#### 3.5.1 Influence of age, sex

Three studies examined the effect of age on duloxetine blood levels, however, none of them found a significant correlation (Table 3). As a reduced kidney function can frequently be found in elderly patients, creatinine clearance was examined by Rovera and colleagues (Rovera et al., 2016). Yet, no significant correlation between creatinine clearance and duloxetine blood levels was found (Rovera et al., 2016). Five studies examined the effect of sex on duloxetine blood levels. None found a significant correlation (De Donatis et al., 2019; Waldschmitt et al., 2009; Volonteri et al., 2010; Rovera et al., 2016; Paulzen et al., 2011).

#### 3.5.2 Influence of smoking

Two studies reported an effect of smoking on duloxetine blood levels (Table 3). Augustin and colleagues (Augustin et al., 2018) found significantly lower median duloxetine blood levels (38.4% lower, *p* = 0.002) and significantly lower C/D ratios (53.6% lower, *p <* 0.001) in smokers than in non-smokers. Additionally, significantly higher daily doses of duloxetine were applied to smokers in comparison to non-smokers (*p* = 0.001) (Augustin et al., 2018). Although no statistical analysis of significance was performed, lower duloxetine blood levels and C/D ratios in smokers compared to non-smokers were also reported by Fric and colleagues (Fric et al., 2008) at two different time points.

#### 3.5.3 Influence of comedication

Two studies examined the influence of comedication on the duloxetine metabolism (Table 3). Paulzen and colleagues (Paulzen et al., 2011) reported a 3.7-fold (statistically significant) increase of C/D ratios under the co-administration of fluvoxamine, a potent inhibitor of CYP1A2. A mean C/D ratio of 1.39 (ng/mL)/(mg/day) was found when co-administering 25 mg fluvoxamine per day in comparison to a mean C/D ratio of 0.37 (ng/mL)/(mg/day) under duloxetine monotherapy (Paulzen et al., 2011). Waldschmitt and colleagues (Waldschmitt et al., 2009) showed significantly lower C/D ratios in patients concomitantly treated with lithium, clozapine and mirtazapine than in patients under duloxetine monotherapy, whereas no significant decrease of C/D ratios was found for co-medication with trimipramine, diazepam, olanzapine and risperidone. In contrast, an increase (not significant) of duloxetine C/D ratio in concomitant treatment with quetiapine and pipamperone was found (Waldschmitt et al., 2009).

### 3.6 Concentration/dose relationship

Five studies reported a linear correlation between duloxetine dose and blood levels (Table 3). In a linear regression analysis, mean doses did not correlate with mean concentrations across 6 studies (*p* = 0.27) (De Donatis et al., 2019; Waldschmitt et al., 2009; Paulzen et al., 2011, 2016; Augustin et al., 2018; Fric et al., 2008). As mean doses and mean blood levels were reported for different time points, the studies by Rovera and colleagues (Rovera et al., 2016) and Volonteri and colleagues (Volonteri et al., 2010) were excluded from linear regression analysis. Mean C/D ratios across one prospective and one retrospective cohort study in patients with different psychiatric diagnoses ranged between 0.37 and 1.38 (ng/mL)/(mg/day) (Paulzen et al., 2011, 2016). In smokers, mean C/D ratios across two studies carried out in a naturalistic setting with different psychiatric diagnoses ranged between 0.27 (Fric et al., 2008) and 0.38 (Augustin et al., 2018), whereas in non-smokers the C/D ratio ranged between 0.81 (Fric et al., 2008) and 0.85 (Augustin et al., 2018). Table 4 presents expected duloxetine blood levels from 30 to 120 mg/d oral doses in somatically healthy populations calculated with mean C/D ratio 0.43 (ng/mL)/(mg/day) (Hiemke et al., 2018).

**Table 4:** Expected duloxetine concentrations from mean C/D ratios after the administration of 30 mg, 60 mg, 90 mg, 120 mg.

	Duloxetine dose per day			
	30 mg	60 mg	90 mg	120 mg
Expected duloxetine blood level from mean (±SD) C/D ratio 0.43 [0.28, 0.58] (ng/mL)/(mg/day)	12.9 [8.4, 17.4] ng/mL	25.8 [16.8, 34.8] ng/mL	38.7 [25.2, 52.2] ng/mL	51.6 [33.6, 69.6] ng/mL

C/D = Concentration-Dose ratio; SD = Standard Deviation.

### 3.7 Population-based target concentration range for duloxetine

**Fig. 1:**
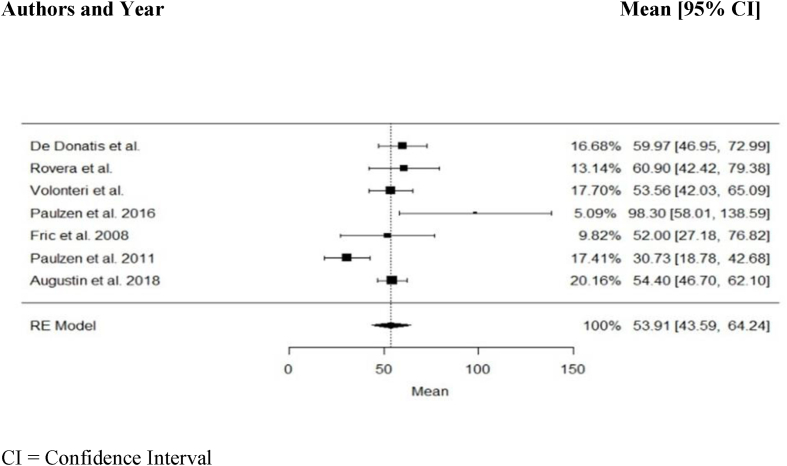
Combined duloxetine mean concentration across 7 studies (*n* = 331).

**Fig. 2:**
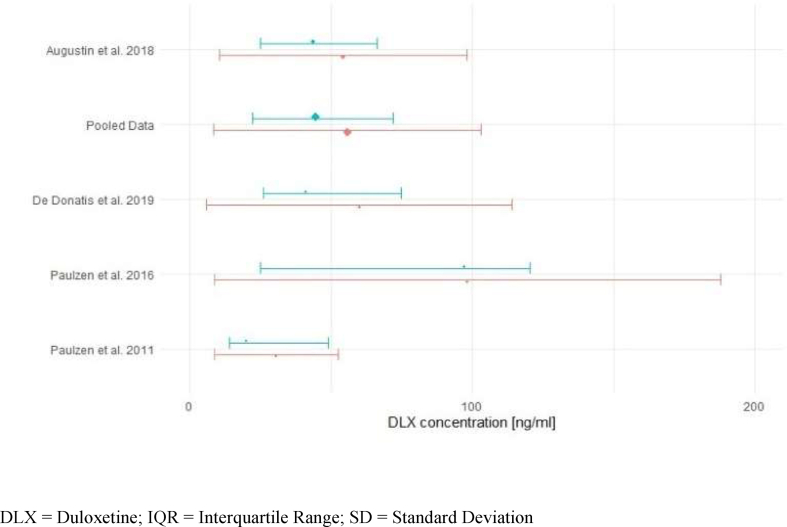
Combined duloxetine median/mean concentration across 4 studies (*n* = 223; median (IQR): 44 (22–72) ng/ml in blue; mean (±SD): 56 (9–103) ng/ml in red). (For interpretation of the references to colour in this figure legend, the reader is referred to the Web version of this article.)

Quantitative analysis was performed using seven studies (*n* *=* 331) from 11 available. Of the 11 studies, three studies were excluded due to a healthy sample cohort (Takano et al., 2006; Abanades et al., 2011; Moriguchi et al., 2017), and one study was excluded as multiple samples were available for several patients (Waldschmitt et al., 2009). The meta-analysis was conducted using a random-effects model. Test for heterogeneity revealed significant differences across the studies (*p* *<* 0.01). Across the 7 studies a mean dose of 70 mg/day resulted in a combined mean duloxetine concentration of 54 ng/mL, 95% CI [44; 64] ng/mL (*n* *=* 331, *Q* *=* 20.02, *p* *<* 0.01*, I*^*2*^ *=* 71.6%, *τ*^*2*^ = 122.2) in adults (Fig. 1). The combined mean ± SD ranged from 7 to 101 ng/mL. A subgroup analysis of moderating factors on mean duloxetine blood levels could be performed with four predefined quality assessment criteria, since at least two studies per subgroup were available. Subgroup comparisons of "diagnosis depression”, “dose design”, and "trough levels" showed no significantly different mean drug concentrations between groups. The model for CYP and psychiatric comedication showed a significant *p*-value in the Test of Moderators. Studies allowing for the use of potentially interfering comedication showed in general a lower mean blood level (38 ng/mL, 95% CI [18; 59] ng/mL) when compared with studies excluding patients with relevant comedication (57 ng/mL, 95% CI [51; 62] ng/mL) (*p* < 0.01) (Supplement Fig. S9). Data for median and IQR was available from four studies (*n* *=* 223). The combined median was 44 ng/mL with an IQR between 22 and 72 ng/mL (mean dose 76 mg/day, mean concentration 56 ng/mL*, p* *=* 0.0003*, I*^*2*^ *=* 90.5%*, Q* = 18.92, *τ* ^*2*^ = 406.4 (SE 403.57)) (Fig. 2). Two studies reported median and interquartile blood levels from responders treated with duloxetine (De Donatis et al., 2019; Waldschmitt et al., 2009). Interquartile ranges (median) of responders were 72–116 (92) ng/mL (De Donatis et al., 2019), and 65–123 (93) ng/mL (Waldschmitt et al., 2009), respectively, and considerably higher than those of non-responders in both studies.

## 4 Discussion

The present work systematically evaluated the relationship between duloxetine blood levels and clinical effects/adverse effects as a basis for its therapeutic reference range. Our qualitative analysis revealed a low level of evidence (Level C) for the relationship between duloxetine blood levels and clinical effects as well as a low level of evidence (Level C) for a relationship between duloxetine blood levels and adverse effects.

### 4.1 Therapeutic reference range for duloxetine

**Fig. 3:**
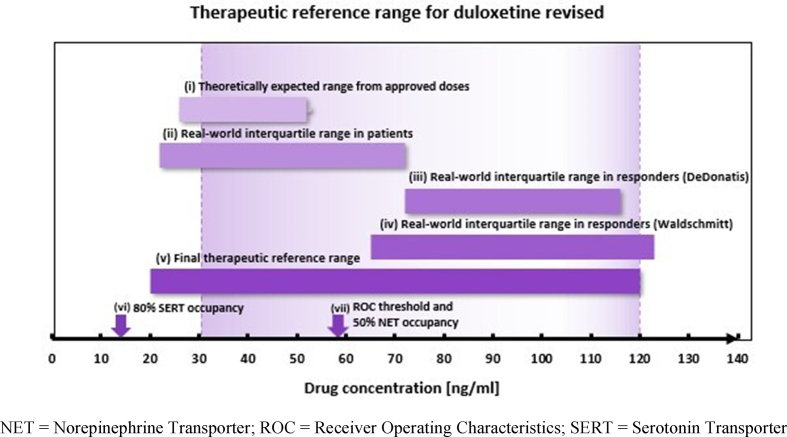
Summary of findings for duloxetine's reference range.

In order to estimate duloxetine's therapeutic reference range, blood levels were calculated for somatically healthy populations and for real-world patients. In somatically healthy populations, expected blood levels range from 26 to 52 ng/mL after the administration of approved doses (60–120 mg) (Table 4). Comparable duloxetine blood levels (22–72 ng/mL) were observed in fifty percent of patients with depression and related disorders treated under flexible and fixed doses across four studies (*n* *=* 223) (De Donatis et al., 2019; Paulzen et al., 2011, 2016; Augustin et al., 2018) (Fig. 2). Blood levels in therapy responders (>50% reduction of HAMD-21 after 3 months) were reported to be considerably higher (IQR 72–116 ng/mL, *n* *=* 17) than in non-responders in a prospective cohort study with fixed doses (De Donatis et al., 2019). Likewise, higher blood levels (IQR 65–123 ng/mL) were found in a TDM survey comparing responders (CGI = 1) with non-responders (CGI ≥2) (Waldschmitt et al., 2009). Both studies reported a positive relationship between blood levels and antidepressant effects (De Donatis et al., 2019; Waldschmitt et al., 2009). Waldschmitt and colleagues (Waldschmitt et al., 2009) additionally revealed a threshold concentration of 58 ng/mL (*p* = 0.011) for clinical response in patients under flexible dosing monotherapy. De Donatis and colleagues (De Donatis et al., 2019) suggested that blood levels higher than 35 ng/mL to a maximum of 120 ng/mL might be needed to obtain the maximum antidepressant effect; blood levels higher 120 ng/mL were not associated with a better antidepressant effect. A third study by Volonteri and colleagues (Volonteri et al., 2010) reported a significant quadratic relationship between blood levels and clinical improvement on anxiety symptoms (HAMA scores) with an optimal anxiolytic effect at duloxetine blood levels of 40–100 ng/mL. In addition, a significant association between the occurrence of irritability/anxiety and the highest duloxetine blood levels (range 5–135 ng/mL) was found (Volonteri et al., 2010). Two PET studies reported that 80% SERT occupancy can be reached with blood levels above 10–15 ng/mL (Takano et al., 2006; Abanades et al., 2011), whereas 50% NET occupancy is reached with blood levels above 58 ng/mL (Moriguchi et al., 2017). The results suggest that in some patients SERT occupancy at low blood levels (10–15 ng/mL) may be sufficient for clinical effects, while others require additional NET action at higher duloxetine blood levels (above 58 ng/mL) to achieve optimal antidepressant effects. In conclusion, we recommend a therapeutic reference range of 20–120 ng/mL for the antidepressant effect of duloxetine in adults (Fig. 3). The lower level reflects the expected concentration from the lowest dose (60 mg/day) recommended as initial and maintenance therapy in real-world patients. The upper threshold is based on the 75th interquartile concentration in responders and rather indicates an optimum in antidepressant response than decreased tolerability. More than 75% of the patients included in our meta-analysis showed drug blood levels below 120 ng/mL. At higher blood levels, increased occurrences of adverse effects such as irritability/anxiety can be expected. However, in case of good clinical response and tolerance, measured blood levels exceeding 120 ng/mL do not necessarily require dose reduction.

### 4.2 Moderating factors and implications for TDM

In contrast to the mean C/D ratio reported in the major international TDM guideline (0.43 (ng/mL)/(mg/day)) (Hiemke et al., 2018), mean C/D ratios ranging from 0.37 to 1.38 (ng/mL)/(mg/day) were reported in two different trials (Paulzen et al., 2011, 2016). A heterogeneous distribution of data concerning the concentration/dose relationship could also be observed across 6 studies for which a clear relationship could not be established (*p* = 0.27). This is not least attributable to varying factors like smoking and comedication in the sample composition (Table 3). In smokers, mean C/D ratios ranged between 0.27 (Fric et al., 2008) and 0.38 (Augustin et al., 2018), whereas in non-smokers mean C/D ratios were considerably higher (0.81 (Fric et al., 2008) and 0.85 (Augustin et al., 2018)). In addition, lower mean blood levels (38 ng/mL) were found in studies allowing for the use of potentially interfering comedication in comparison to studies excluding patients with relevant comedication (57 ng/mL) (*p* < 0.01) (Supplement Fig. S9). Smoking and potentially interfering comedication were identified as clinically relevant factors on duloxetine blood levels and most likely impact the effectiveness of duloxetine treatment (Table 3). As PET studies have shown, optimal clinical effects may occur at low blood levels (10–15 ng/mL) through SERT occupancy (Takano et al., 2006; Abanades et al., 2011). However, some patients require additional NET action at higher duloxetine blood levels (above 58 ng/mL) (Moriguchi et al., 2017). Hence, drug level guided dosing is particularly indicated for patients who have been newly prescribed with duloxetine due to several factors that can affect blood levels.

A similar study for the SNRI venlafaxine also found that TDM is recommended upon initial prescription (Lense et al., 2023). Factors such as sex, age, specific comedication, and CYP2D6/CYP2C19 metabolizer status were reported to influence the effectiveness of venlafaxine treatment through altered blood levels. Additionally, while low blood levels were associated with SERT occupancy, considerably higher blood levels were needed for occupancy of the NET (Lense et al., 2023; Hart et al., 2024). Clinical effects may occur throughout a broad concentration range due to SNRI's inhibition of both SERT and NET. However, confirmation from future studies is needed.

### 4.3 Limitations

Similar studies on therapeutic reference ranges exist for other psychotropic drugs (Lense et al., 2023; Hart et al., 2022). Despite the limited number of studies available, a high quality of TDM was found in most studies allowing for the derivation of a well-established therapeutic reference range. As it has recently been shown, the concentration-effect relationship is significantly moderated by the exclusion of blood levels in lower- and subtherapeutic ranges (Funk et al., 2022). Noteworthy, sufficiently broad (sub- and/or supratherapeutic) blood level ranges were included in all concentration-effect studies assessed within this review (Supplement Table S6). Nevertheless, there are limitations to the quality of the recommended reference range, such as the designs of the studies supporting it. Study designs presented in this work varied and data was mostly extracted from naturalistic and small non-controlled studies. Naturalistic studies typically allow for flexible dosing. However, Funk and colleagues (Funk et al., 2022) showed that flexible dosing is a significant influencing factor leading to artificial findings of concentration-effect relationships. This is, for example, attributed to the frequent dose adjustments made in flexible dose studies, driven by non-response or the occurrence of adverse effects. These adjustments contribute to an improvement in the drug's efficacy when compared to the control group (Funk et al., 2022). A systematic difference between fixed and flexible dosing has also been seen in a meta-analysis that found significantly higher mean blood levels in fixed dose studies compared to flexible dose studies (Hart et al., 2022). There are further limitations to naturalistic studies, such as the potential inclusion of patients with multiple diagnoses in the study, the use of psychiatric comedication and interventions, and the likelihood for systemic over- or underdosing in specific patient groups. Collecting samples at trough blood levels, achieving steady state, or ensuring an equal number of samples across the patient population cannot always be guaranteed in naturalistic studies and may result in a misinterpretation of the acquired data. In addition, high quality randomized controlled trials investigating concentration-effect relationships and studies using a placebo lead-in phase were missing to support the target range for duloxetine. The use of a quadratic function to depict a concentration-effect relationship is another limitation found (Zernig et al., 1996, 2020). It has been shown by Zernig and colleagues (Zernig et al., 1996, 2020) that rather a logistic function than a quadratic function should be used to fit the concentration-effect relationship. The present work aimed at providing a comprehensive overview of the effects and safety of duloxetine regarding blood level ranges. As our primary focus was the relationship between blood levels and clinical effects/adverse effects, dose/response studies were not considered although they could have provided additional insights. The therapeutic reference range investigated in this review is restricted to adult patients. To extend it to children/adolescent or elderly patients, additional prospective studies involving these age groups are necessary.

## 5 Conclusion

We recommend a target duloxetine blood level of 20–120 ng/mL to achieve antidepressant effects in adults. The lower level indicates a threshold for antidepressant response and subtherapeutic blood levels may lead to poor response. Some patients will benefit at low doses predominantly from duloxetine's SERT occupancy. Other patients, however, may require higher doses to achieve sufficient NET occupancy. In case of non-response at low to medium concentrations (i.e. < 60 ng/mL), we recommend dose titration within the proposed reference range. Attaining blood levels within 20–120 ng/mL increases the probability of response while the risk of adverse effects is kept to a minimum. The blood level of 120 ng/mL marks the upper threshold of the reference range. It reflects an optimum in antidepressant response; however, the risk for adverse effects increases with higher, particularly with supratherapeutic blood levels. Good clinical response and tolerance given, blood levels exceeding 120 ng/mL do not necessarily require dose reduction. The effectiveness of duloxetine treatment is likely influenced by smoking and specific comedications through altered blood concentrations. This warrants a special indication for TDM of duloxetine, particularly when newly prescribed.

## Funding statement

This research did not receive any specific grant from funding agencies in the public, commercial, or not-for-profit sectors.

## Data availability statement

Generated Statement: The original contributions presented in the study are included in the article/supplementary material, further inquiries can be directed to the corresponding author/s.

## Author contributions

FA developed the first draft of the protocol. XMH and GG supervised the entire manuscript writing and contributed to the revision of the protocol. XMH, FA, and GG contributed to the development of the search strategy. FA and XMH contributed to the quality assessment. FA, XMH, GG, GZ and MK confirmed grading of the level of revealed evidence. All authors have read and approved the final manuscript.

## Declaration of competing interest

The authors declare the following financial interests/personal relationships which may be considered as potential competing interests: F. Amann reports a relationship with AbbVie Deutschland GmbH & Co KG Ludwigshafen that includes: employment. Dr. G. Gründer reports a relationship with Allergan that includes: consulting or advisory. Dr. G. Gründer reports a relationship with Boehringer Ingelheim GmbH that includes: consulting or advisory and funding grants. Dr. G. Gründer reports a relationship with Institute for Quality and Efficiency in Health Care that includes: consulting or advisory. Dr. G. Gründer reports a relationship with Janssen-Cilag that includes: consulting or advisory and speaking and lecture fees. Dr. G. Gründer reports a relationship with Lundbeck that includes: consulting or advisory, funding grants, and speaking and lecture fees. Dr. G. Gründer reports a relationship with MindMed that includes: consulting or advisory. Dr. G. Gründer reports a relationship with Otsuka that includes: consulting or advisory and speaking and lecture fees. Dr. G. Gründer reports a relationship with Recordati that includes: consulting or advisory and speaking and lecture fees. Dr. G. Gründer reports a relationship with Roche that includes: consulting or advisory. Dr. G. Gründer reports a relationship with ROVI that includes: consulting or advisory. Dr. G. Gründer reports a relationship with Sage that includes: consulting or advisory. Dr. G. Gründer reports a relationship with Takeda that includes: consulting or advisory. Dr. G. Gründer reports a relationship with Gedeon Richter that includes: speaking and lecture fees. Dr. G. Gründer reports a relationship with Beckley Psytech that includes: funding grants. Dr. G. Gründer reports a relationship with Mind and Brain Institute GmbH that includes: board membership. Dr. G. Gründer reports a relationship with Brainfoods GmbH that includes: board membership. Dr. G. Gründer reports a relationship with OVID Health Systems GmbH that includes: board membership. Dr. G. Gründer reports a relationship with MIND Foundation gGmbH that includes: board membership. Dr. X. Hart reports a relationship with 10.13039/501100001691Japan Society for the Promotion of Science that includes: funding grants. Dr. G. Gründer serves as Editorial Board Member for Neuroscience Applied If there are other authors, they declare that they have no known competing financial interests or personal relationships that could have appeared to influence the work reported in this paper.
