# Supplementary material for: Therapeutic reference range for duloxetine in the treatment of depression revised: A systematic review and meta-analysis
Source: Neurosci Appl. 2024 Jun 10;3:104077. doi: 10.1016/j.nsa.2024.104077 (PMC12244135; doi:10.1016/j.nsa.2024.104077)
Supplement: Multimedia component 1 [file mmc1.pdf]

Data Supplement
"Therapeutic reference range for duloxetine in the treatment of depression revised: a systematic review and meta-analysis"

F. Amann[1], M. Kochtyrev[2], G. Zernig[2,3,4], G. Gründer[1,4,6], X.M. Hart[1,4,5]

[1]*Central Institute of Mental Health, Department of Molecular Neuroimaging, Medical Faculty Mannheim, University of Heidelberg, Mannheim, Germany.*

[2]*Medical University of Innsbruck, Department of Pharmacology, Innsbruck, Austria.*

[3]*Private Practice for Psychotherapy and Court-Certified Witness, Hall in Tirol, Austria.*

[4]*Arbeitsgemeinschaft für Neuropsychopharmakologie und Pharmakopsychiatrie (AGNP), Working Group "Therapeutic Drug Monitoring"*

[5]*Department of Neuropsychiatry, Keio University School of Medicine, Tokyo, Japan*

[6]*German Center for Mental Health, partner site Mannheim – Heidelberg – Ulm, Germany*

S1. Full database search strings

PubMed

("Duloxetine Hydrochloride"[Mesh] OR "Duloxetine"[tiab]) AND ("serum level*"[tiab] OR "plasma level*"[tiab] OR "blood level*"[tiab] OR "drug level*"[tiab] OR "serum concentration*"[tiab] OR "plasma concentration*"[tiab] OR "blood concentration*"[tiab] OR "drug concentration*"[tiab] OR "Drug Monitoring"[Mesh] OR "drug monitor*"[tiab] OR "positron emission tomography"[Mesh] OR "positron emission tomograph*"[tiab] OR "PET scan*"[tiab] OR "Tomography, Emission Computed, Single-Photon"[Mesh] OR "single photon emission*"[tiab] OR "SPECT"[tiab] OR "CAT scan*"[tiab] OR "CT scan*"[tiab]) NOT ("Animals"[MeSH Terms] NOT "humans"[MeSH Terms])

Web of Science Core Collection

TS=("Duloxetine") AND TS=("serum level*" OR "plasma level*" OR "blood level*" OR "drug level*" OR "serum concentration*" OR "plasma concentration*" OR "blood concentration*" OR "drug concentration*" OR "drug monitor*" OR "positron emission tomograph*" OR "PET scan*" OR "single photon emission*" OR "SPECT" OR "CAT scan*" OR "CT scan*")

Cochrane Library

(Duloxetine) AND (((serum OR plasma OR blood OR drug) NEXT (level* OR concentration*)) OR (drug NEXT monitor*) OR (positron NEXT emission NEXT tomograph*) OR (PET NEXT scan*) OR (single NEXT photon NEXT emission*) OR SPECT OR (CAT NEXT scan*) OR (CT NEXT scan*))

PsycINFO

("Duloxetine") AND ("serum level*" OR "plasma level*" OR "blood level*" OR "drug level*" OR "serum concentration*" OR "plasma concentration*" OR "blood concentration*" OR "drug concentration*" OR "drug monitor*" OR "positron emission tomograph*" OR "PET scan*" OR "single photon emission*" OR "SPECT" OR "CAT scan*" OR "CT scan*")

S2. Study Overview according to PRISMA

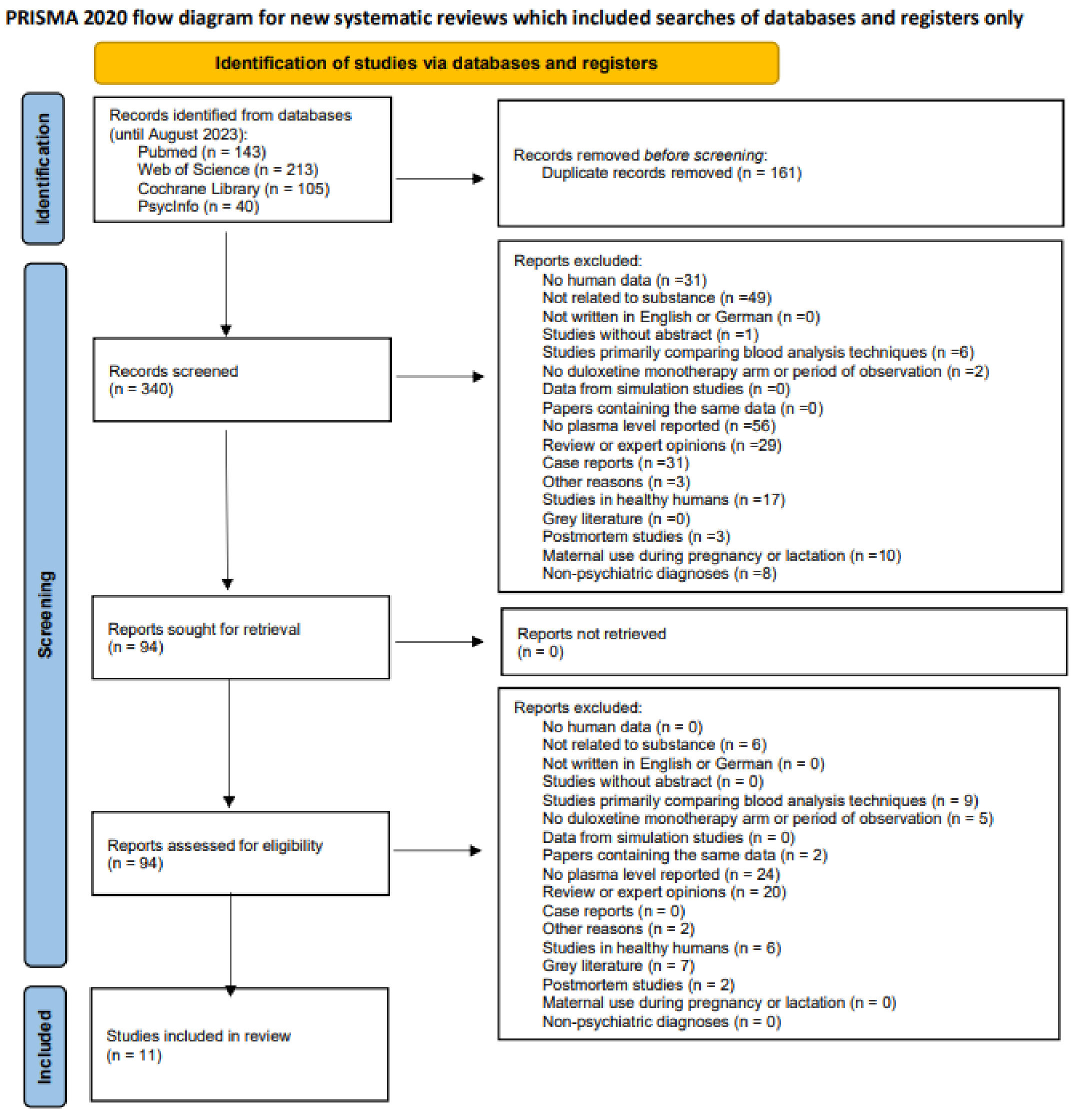

From: Page MJ, McKenzie JE, Bossuyt PM, Boutron I, Hoffmann TC, Mulrow CD, et al. The PRISMA 2020 statement: an updated guideline for reporting systematic reviews. BMJ 2021;372:n71. doi: 10.1136/bmj.n71

For more information, visit: http://www.prisma-statement.org/

Table S3. Inclusion and exclusion criteria for study eligibility

	Inclusion criteria	Exclusion criteria
**Population**	– Psychiatric patients treated with duloxetine[B]<br>– Indications for treatment are depressive disorders[B]	– Non-human subjects<br>– Healthy volunteers, non-psychiatric patients[B]<br>– Postmortem studies<br>– Maternal use during pregnancy or lactation
**Intervention**	– Duloxetine monotherapy arm or period of observation (at least one blood level measurement before add-on therapy)<br>– Treatment duration long enough to reach steady state (3 days)[B]	– Blood level is not measured in the steady state[B]<br>– Studies primarily comparing blood analysis techniques
**Outcome(s)**	– Drug concentrations measured in the blood (serum or plasma)<br>– For concentration-effect studies: direct clinical outcome measures are reported, i.e., safety or efficacy using a standardized rating scale (e.g., HAMD, MADRS, CGI)[A]<br>– For neuroimaging studies: serotonin receptor and/or norepinephrine receptor occupancy	– No mean or median blood level reported
**Study Design**	– observational and interventional studies are included<br>– Reviews and meta-analyses investigating a concentration-effect relationship for duloxetine	– Reviews and experts' opinions<br>– Grey literature<br>– Case reports and case series
**Other**		– Papers containing the same data<br>– No abstract available<br>– Not written in English or German<br>– Data from simulation studies

[A] Biomarkers (e.g. QTc-time) are not regarded a direct clinical outcome measure.

[B] Not applicable for neuroimaging studies.

Table S4. Detailed information on concentration-effect studies and concentration studies

**Author, year**	**Country**	**Study Design**	**Subjects**	**Mean DLX dose ± SD (range) [mg/day]**	**Mean DLX BL ± SD (Range) [ng/mL]**	**Median DLX BL (IQR) [ng/mL]**	**Comment / Main outcome**
**Concentration-effect studies**							
De Donatis et al. 2019	Italy	Prospective CS with fixed doses in outpatients	*n*=66, MDD (HAMD-21 ≥ 14);<br>39.4% males;<br>age 56.42 ± 14.55	60	59.96 ± 53.96<br>(3-320)	41.0<br>(26-75)	Curvilinear (quadratic) relationship between DLX BLs and antidepressant response after excluding patients with BLs lower than 35 ng/mL. Maximum antidepressant effect between 35 and 120 ng/mL.<br>IQR of responders: 72-116 ng/mL; IQR of non-responders: 18-50 ng/mL<br>No adverse effects reported.
Rovera et al. 2016	Italy	Prospective CS with flexible doses in outpatients in naturalistic setting	*n*=35, MDD (DSM-IV);<br>17.1% males;<br>age 78.91 ± 5.14	T0:<br>39.70 ± 19.15	1 month:<br>60.90 ± 55.77<br>(4.9-201.9)	N/A	Patients aged ≥ 65 years.<br>Significant negative correlation between DLX BLs and the percentage of improvement of BDI- ($p < 0.001$) and HAMA scores ($p < 0.05$) at 12 months.<br>No significant relationship between plasma levels and HAMD-21 improvement.<br>No relationship between DLX BLs and adverse effects
Volonteri et al. 2010	Italy	Prospective CS with flexible doses in outpatients in naturalistic setting	*n*=45, MDD (DSM-IV),<br>35.6% males;<br>age 59.6 ± 12.79	T0:<br>58.0 ± 7.56	1 month:<br>53.56 ± 39.45<br>(5-135)	N/A	No significant correlation between HAMD-21 scores and DLX BLs found (R^2=0.05; p=0.502).<br>Significant curvilinear (quadratic) relationship between DLX BLs and clinical improvement on anxiety symptoms (HAMA scores) with a maximum at DLX BLs between 40 and 100 ng/mL.<br>Significant association between the occurrence of irritability/anxiety and higher DLX BLs.
Waldschmitt et al. 2009	Germany	Retrospective TDM CSS with flexible doses in naturalistic setting	*n*=103, F31-F34,<br>31.1% males;<br>age 54.9	80 ± 26<br>(30-120)	58<br>(2-318)	37 (23-66)	In patients under DLX monotherapy (*n*=36), higher BLs were identified in responders (CGI=1) than in non-responders (CGI ≥ 2) with ROC threshold concentration for good clinical response at 58 ng/mL (p=0.011).<br>Median of responders: 93 ng/mL (IQR: 65-123); Median of non-responders: 47 ng/mL (23-54).<br>Mean BL of 60 ng/mL in patients with mild adverse effects vs. mean BL of 56 ng/mL in patients with no adverse effects. Multiple blood samples available for several patients.

Author, year	Country	Study Design	Subjects	Mean DLX dose ± SD (range) [mg/day]	Mean DLX BL ± SD (Range) [ng/mL]	Median DLX BL (IQR) [ng/mL]	Comment / Main outcome
**Concentration studies**							
Paulzen et al. 2016	Germany	Prospective CS with fixed doses	*n*=19, multiple Dx; 42.1% males; age 63.2 ± 13.7	71.1 ± 30.3 (30-120)	98.3 ± 89.6 (2.4-323)	N/A	Blood sampling not at trough; DLX serum concentration moderately correlated with DLX dose (r=0.473; p=0.04).
Fric et al., 2008	Germany	Prospective CS with flexible doses	*n*=28, multiple Dx; 75% males; age 47.3 ± 14.4	90 ± 19 (60-120)	52 ± 67 (6-325)	N/A	Lower DLX BLs and C/D ratios in smokers than in non-smokers at two different time points. Doses were higher in smokers than in non-smokers. No statistical analysis of significance.
Paulzen et al. 2011	Germany	Retrospective CS with flexible doses	*n*=13, MDD; 23.1% males; age 61.15 ± 12.35	87.7 ± 14.8 (30-120)	30.7 ± 21.98 (2-72.8)	20.1 (14-49.15)	3.7-fold (statistically significant) increase of C/D ratios under the co-administration of fluvoxamine. A mean C/D ratio of 1.39 (ng/mL)/(mg/day) was found when co-administering 25 mg fluvoxamine per day in comparison to a mean C/D ratio of 0.37 (ng/mL)/(mg/day) under DLX monotherapy.
Augustin et al. 2018	Germany	Retrospective TDM CSS with flexible doses	*n*=125, multiple Dx; 27.2% males; age 57.42	82.56 ± 26.58 (30-150)	54.4 ± 43.9 (5.7-230)	43.8 (25.10-66.40)	Significantly lower median DLX BLs (38.4% lower, p=0.002) as well as significantly lower C/D ratios (53.6% lower, p<0.001) in smokers than in non-smokers. Smokers received higher daily doses of DLX (p=0.001).

Table S5. PET studies reporting SERT / NET occupancy and duloxetine blood concentrations

**Author, year**	**PET tracer**	**Study Design**	**Subjects**	**Exclusion criteria**	**Mean DLX dose (range) [mg/day]**	**Mean DLX BL ± SD (range) [ng/mL]**	**Mean receptor occupancy [%]**	**EC_{50} [ng/mL]**	**SERT EC_{80} [ng/mL]**	**Comment / Main outcome**
Takano et al. 2006	[11C]DASB	Prospective CS; 12 participants with single DLX administration (5, 20, 40 or 60 mg), 3 participants with repeated DLX administration (60 mg for 7 consecutive days); trough samples analysed by LC-MS/MS	*n*=15, healthy volunteers; mean age 24.1 ± 2.4; 100% males	N/A	31.3 ± 21.7 (5-60) (after single dosing)	N/A	69.3 ± 16.5 (after single dosing)	3.7 (SERT) (single dosing)	15.0 (single dosing)	Mean occupancies after single dosing were 43.6 ± 8.8% at 5 mg, 71.3 ± 5.3% at 20 mg, 80.6 ± 4.8% at 40 mg, and 81.8 ± 4.3% at 60 mg. Good relationship between DLX BLs and SERT occupancy with ED_{50}=7.9 mg and EC_{50}=3.7 ng/mL. Doses higher 40 mg are necessary to reach 80% SERT occupancy. Maintenance of high SERT occupancy levels after multiple dosing of 60 mg, even after decreasing DLX BLs.
Abanades et al. 2011	[11C]DASB	Prospective CS; administration of single dose of 20 mg DLX followed by a 20 mg dose on 4 consecutive days; trough samples analysed by HPLC-MS/MS	*n*=10, healthy volunteers; mean age 40.2 ± 11.1; 100% males	Alcohol or illicit substance abuse or dependence Serious medical or neurologic illness, current or lifetime history of DSM-IV Axis I or II diagnosis and/or suicidal tendencies	20.0	19.74 ± 7.8 (5.98-34.17)	78.2 ± 5.1	2.6 ± 0.93 (SERT)	10.5	Average occupancy (across the midbrain, striatum and thalamus) of 78.2 ± 4.9% was measured after repeat dosing with a mean DLX BL of 19.74 ± 7.8 ng/mL. An indirect PK/PD model describing the relationship between DLX BLs and SERT occupancy after single dosing estimated an EC_{50} concentration of 2.6 ± 0.93 ng/mL.
Moriguchi et al. 2017	(S,S)-[18F]FMeNER-D_2	Prospective CS; single doses of 20, 40 or 60 mg; trough samples analysed by GC-MS	*n*=8, healthy volunteers; mean age 25.5 ± 5.9; 100% males	Psychiatric, neurological or somatic illness Drug abuse	37.5 ± 16.7 (20-60)	29.44 ± 24 (4.1-80.8)	32.6 ± 9.8	58.0 (NET)	NA	Significant correlation (*r*=0.72, *p*=0.044) between DLX BL (mean 29.44 ± 24 ng/mL) and NET occupancy (mean 32.6 ± 9.8%) with EC_{50} estimated at 58.0 ng/mL and ED_{50} at 76.8 mg.

Table S6. Rating results of general quality criteria for the therapeutic drug monitoring component for all studies[1]

**Reference**	**Selection (max. 3 points)**		**Comparability (max. 2 points)**		**Drug Monitoring (max. 5 points)**			**Total Score (x/10)**
	**Q1**	**Q2**	**Q3**	**Q4**	**Q5**	**Q6**	**Q7**	
a. ***Concentration-effect studies***								
**Cohort studies**								
De Donatis et al. 2019	x	xx	x	x	x	xx	ox	9/10
Rovera et al. 2016	o	xx	x	o	x	xx	ox	7/10
Volonteri et al. 2010	x	xx	x	o	x	xx	ox	8/10
**Cross-sectional studies**								
Waldschmitt et al. 2009	x	xo	x	o	x	xx	ox	7/10
b. ***Concentration studies***								
**Cohort studies**								
Paulzen et al. 2016	x	xo	x	x	x	xo	ox	7/10
Fric et al. 2008	x	xo	x	o	x	xx	ox	7/10
Paulzen et al. 2011	x	xx	o	o	x	xx	xx	8/10
**Cross-sectional studies**								
Augustin et al. 2018	o	oo	x	o	x	xx	ox	5/10
c. ***Neuroimaging studies***								
**Cohort studies**								
Abanades et al. 2011	o	xx	?	x	x	xx	xx	8/10
Moriguchi et al. 2017	o	ox	?	x	x	ox	xx	6/10
Takano et al. 2006	o	ox	x	x	o	xx	xo	6/10

x = item rated sufficient; o = item rated insufficient; ? = no information regarding item given

Q1 = Representativeness of the patient sample; Q2 = Diagnosis; Q3 = Comedication; Q4 = Dose design; Q5 = Analytical method for the assay of drug concentration in serum or plasma; Q6 = Blood sample collection; Q7 = Concentration design

[1]Due to an elimination half-life of approximately 12 hours, steady state is presumed after three days (six elimination half-lives) of constant oral dosing. Sampling should rather reflect a minimum than a maximum concentration in the blood, optimally a time point immediately prior to the next dose. Comedication with CYP1A2 or CYP2D6 inducers or inhibitors potentially alters pharmacokinetics in the study sample. Comedication might plausibly influence DLX pharmacodynamics and thus was covered by quality assessment. Furthermore, polymorphisms in CYP2D6 have been shown to be ethnicity related. Blood level measurements in clinical studies should be performed using a validated method and cover concentrations of 10% around the currently defined lower threshold.

Table S7. Study type specific quality assessment for cohort studies

Reference	Selection (max. 4 points)				Comparability (max. 2 points)	Outcome (max. 4 points)				Total score (x/10)
	Q1	Q2	Q3	Q4	Q5	Q6	Q7	Q8	Q9	
a. Concentration-effect studies										
De Donatis et al. 2019	x	o	o	x	xx	x	x	o	x	7/10
Rovera et al. 2016	o	o	?	x	xo	x	x	o	x	5/10
Volonteri et al. 2010	x	o	?	x	xx	x	x	o	x	7/10
b. Concentration studies										
Paulzen et al. 2016	x	o	o	o	xx	x	x	o	x	6/10
Fric et al. 2008	x	x	o	x	xx	x	x	x	o	8/10
Paulzen et al. 2011	x	x	o	x	xx	x	x	x	x	9/10
c. Neuroimaging studies										
Abanades et al. 2011	o	o	o	x	xx	x	x	x	x	7/10
Moriguchi et al. 2017	o	o	o	x	xx	x	x	x	x	7/10
Takano et al. 2006	o	x	o	x	xx	x	x	x	o	7/10

x = item rated sufficient; o = item rated insufficient; ? = no information regarding item given

Q1 = Representativeness of the exposed cohort; Q2 = Selection of the control cohort; Q3 = Ascertainment of exposure; Q4 = Demonstration that outcome of interest was not present at start of study; Q5 = Comparability of "exposed" and "non-exposed" individuals or of outcome groups; Q6 = Assessment of outcome; Q7 = Was follow up long enough for outcomes to occur; Q8 = Adequacy of follow up of cohorts; Q9 = Statistical tests

Table S8. Study type specific quality assessment for cross-sectional studies

Reference	Selection (max. 4 points)				Comparability (max. 2 points)	Outcome (max. 2 points)		Total score (x/8)
	Q1	Q2	Q3	Q4	Q5	Q6	Q7	
a. Concentration-effect studies								
Waldschmitt et al. 2009	x	o	o	o	xx	x	x	5/8
b. Concentration studies								
Augustin et al. 2018	o	?	x	x	xx	x	o	5/8

x = item rated sufficient; o = item rated insufficient; ? = no information regarding item given

Q1 = Representativeness of the sample; Q2 = Sample size; Q3 = Non-respondents; Q4 = Ascertainment of exposure; Q5 = Comparability of outcome groups; Q6 = Assessment of outcome; Q7 = Statistical tests

Figure S9. Overall mean duloxetine concentration estimate [ng/mL] with subgroup analysis "comedication" (n=331)

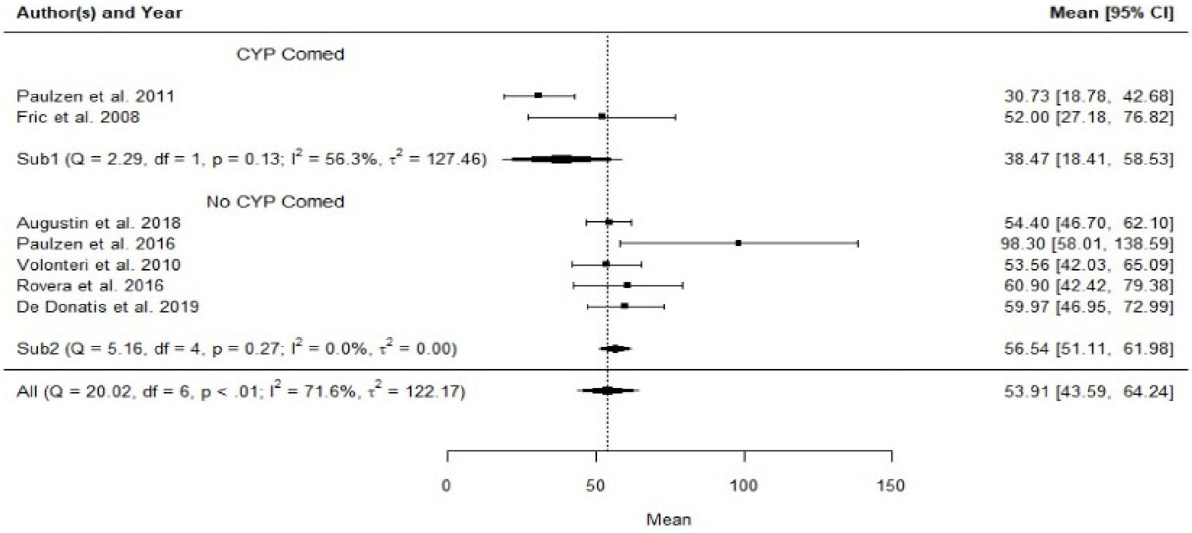

S10. Abbreviations used in the data supplement

BDI	Beck Depression Inventory
BL	Blood Level
CAT	Computer-assisted Tomography
C/D	Concentration Dose ratio
CGI	Clinical Global Impression
CS	Cohort Study
CSS	Cross-sectional study
CT	Computed Tomography
CYP	Cytochrome P450
DLX	Duloxetine
DSM-IV	Diagnostic and Statistical Manual of Mental Disorders
Dx	Medical Diagnosis
EC_{50}	Effective Concentration associated with 50% transporter occupancy
EC_{80}	Effective Concentration associated with 80% transporter occupancy
ED_{50}	Effective Dose associated with 50% transporter occupancy
GC-MS	Gas Chromatography Mass Spectrometry
HAMA	Hamilton Rating Scale for Anxiety
HAMD	Hamilton Rating Scale for Depression
HAMD-21	Hamilton Rating Scale for Depression, 21-item version
HPLC-MS/MS	High Performance Liquid Chromatography Mass Spectrometry/Mass Spectrometry
IQR	Interquartile Range
LC-MS/MS	Liquid Chromatography-Mass Spectrometry/Mass Spectrometry
MADRS	Montgomery–Åsberg Depression Rating Scale
MDD	Major Depressive Disorder
mg	milligram
mL	milliliter
n	number of subjects treated with duloxetine
N/A	Not Available
NET	Norepinephrine Transporter
ng	nanogram
PET	Positron Emission Tomography
PK/PD	Pharmacokinetic/Pharmacodynamic
PRISMA	Preferred Reporting Items for Systematic Reviews and Meta-Analysis
QT_c	corrected QT Interval
ROC	Receiver Operating Characteristics
SD	Standard Deviation
SERT	Serotonin Transporter
SPECT	Single Photon Emission Computed Tomography
TDM	Therapeutic Drug Monitoring
